# Updated regulation curation model at the *Saccharomyces* Genome Database

**DOI:** 10.1093/database/bay007

**Published:** 2018-02-27

**Authors:** Stacia R Engel, Marek S Skrzypek, Sage T Hellerstedt, Edith D Wong, Robert S Nash, Shuai Weng, Gail Binkley, Travis K Sheppard, Kalpana Karra, J Michael Cherry

**Affiliations:** Department of Genetics, Stanford University, Stanford, CA 94305, USA

## Abstract

The *Saccharomyces* Genome Database (SGD) provides comprehensive, integrated biological information for the budding yeast *Saccharomyces cerevisiae*, along with search and analysis tools to explore these data, enabling the discovery of functional relationships between sequence and gene products in fungi and higher organisms. We have recently expanded our data model for regulation curation to address regulation at the protein level in addition to transcription, and are presenting the expanded data on the ‘Regulation’ pages at SGD. These pages include a summary describing the context under which the regulator acts, manually curated and high-throughput annotations showing the regulatory relationships for that gene and a graphical visualization of its regulatory network and connected networks. For genes whose products regulate other genes or proteins, the Regulation page includes Gene Ontology enrichment analysis of the biological processes in which those targets participate. For DNA-binding transcription factors, we also provide other information relevant to their regulatory function, such as DNA binding site motifs and protein domains. As with other data types at SGD, all regulatory relationships and accompanying data are available through YeastMine, SGD’s data warehouse based on InterMine.

**Database URL**: http://www.yeastgenome.org

## Introduction

The *Saccharomyces* Genome Database (SGD; http://www.yeastgenome.org) collects and organizes scientific information regarding the genes, proteins and other chromosomal features of the model organism *Saccharomyces cerevisiae*, and then makes this knowledge freely available to the public. This information includes regulatory relationships that exist between specific genes and proteins, in which one entity controls some aspect of the expression or enzymatic activity of the other. As with other types of data collected at SGD, the primary source of regulation annotations is research results published in the scientific literature. The availability of large-scale studies has greatly increased the volume of collected regulation data. The update and further integration of these regulatory data into SGD in a coherent and comprehensive manner has been a major focus of our recent activities. Yeast is already a valuable model organism for the study of different types of regulation (1–4). These data will help researchers investigate the regulation of specific cellular functions, as well as entire regulatory cascades, furthering the value of yeast as a widely used model organism.

Regulatory relationships refer to the interactions between genes and proteins that coordinate the expression of genes and activity of proteins. This coordination can occur at many different levels, including transcription, RNA activity, RNA stability, translation, protein activity and protein stability. The sum of regulatory relationships within an organism comprises its regulatory network. By capturing these relationships, SGD is systematically detailing the regulatory network of budding yeast. Cataloguing these regulatory relationships and the different levels at which they occur is a key step toward building a complete understanding of a model regulatory network of a eukaryotic cell. To this end, we have expanded our regulation curation model beyond DNA-binding transcription factors to other chromatin modifiers, and to the regulation of protein activity as well by leveraging our growing body of curated post-translational modifications (PTMs). Data from large-scale screens for RNA expression levels, chromatin immunoprecipitation, or other types of screens that use genomic techniques, were previously used to seed the SGD Regulation pages in an effort to provide broad coverage for as many genes as possible. We have since turned our focus to expert curation of more focused, smaller-scale studies. We describe here the expanded regulation curation model recently put into place at SGD.

## Curation data model

SGD curators survey the yeast literature to find and record regulatory relationships for as many genes as possible in the yeast genome. This activity includes many papers that have characterized specific regulatory relationships and identified the genes and proteins involved, as well as large-scale studies, many of which report on regulation for hundreds of genes at a time. In addition to these targeted regulation curation efforts, SGD curators record regulatory relationships from newly published papers on a weekly basis as part of regular curation activity. We are also leveraging existing Gene Ontology (GO; 5, 6) and PTM annotations (7) to focus and prioritize our activities. Annotations to GO terms such as ‘regulation of gene expression’ (GO: 0010468), ‘cellular protein modification process’ (GO: 0006464) and other similar terms and their descendants aid in the identification of high-value genes and publications for regulation curation. Furthermore, by recording results from the same experiment in different areas of the database (functional data as GO annotations, protein modifications as PTM annotations, regulation data as regulatory relationships), SGD presents as complete of a picture of biology as possible. It is valuable to present the same data in multiple ways in order to serve scientists, educators and students coming from different perspectives that are often interested in varied aspects of cellular biology.

Regulation data are recorded in the database using controlled vocabularies. The regulatory relationships themselves are each recorded as a regulator-target pair. Other pieces of information captured include the type of regulation that is being described, the role of the regulator, the direction of the regulation, the cellular situation under which the regulation occurs, the type of experiment used to investigate the relationship and the publication in which the relationship was described (Table 1). Currently included aspects of regulation include transcription and protein activity; this will be expanded in the future to include RNA stability and protein stability. Regulator types include transcription factors, chromatin modifiers and protein modifiers; regulators are broadly classified based on their GO annotations. In the future, this scope will be increased to include RNA-binding proteins and RNA modifiers. Direction of regulation is described as positive (i.e. activation), negative (i.e. repression or deactivation) or ‘null.’ This latter case is used for experiments that investigate binding sites without demonstrating the outcome of that binding (ex., ChIP-chip). We use existing GO terms to describe the cellular conditions under which the regulation occurs. On Regulation pages, this is listed as ‘happens during,’ and the terms in use include descendants of ‘cellular response to stimulus’ (GO: 0051716) such as ‘cellular response to heat,’ ‘cellular response to hypoxia,’ etc. Also included in ‘happens during’ are the GO terms for cell cycle phases, such as ‘mitotic M phase,’ ‘meiotic G2 phase,’ etc. when relevant.

**Table 1.** Updated curation data model at SGD

Data type	Description	Value
Regulator	Gene identifier	Gene name or systematic name
Regulator type^a^	Controlled vocabulary	Transcription factor
		Chromatin modifier
		Protein modifier
Target	Gene identifier	Gene name or systematic name
Direction	Controlled vocabulary	Positive
		Negative
		null
Regulation of^b^	Controlled vocabulary	Transcription
		Protein activity
Happens during	Controlled vocabulary: Situation under which the regulation occurs	Defined subset of GO terms: descendent terms of Biological Process ‘cellular response to stimulus’ GO: 0051716 and ‘cell cycle phase’ GO: 0022403
Annotation method	Controlled vocabulary	Manually curated
		High-throughput
Evidence	Type of experiment used to test for and/or demonstrate the regulatory relationship	ECO term
Strain background	Controlled vocabulary	Strain name
Reference	Publication in which the regulatory relationship is described	PubMed ID

a Future expansion to include RNA-binding proteins and RNA modifiers.

b Future expansion to include RNA stability and protein stability.

Controlled vocabularies are also used to describe additional aspects of the regulation annotations, including details such as annotation method, assay performed, and strain background. Annotation method includes ‘manually curated’ and ‘high-throughput.’ Results from small-scale experiments that focus on one or a few genes are reviewed completely by curators and presented as ‘manually curated’ annotations. ‘High-throughput’ denotes experiments designed with some knowledge of the genome sequence, such as systematic mutation sets that include collections of deletion strains, or those that use high-throughput, robot-assisted techniques, to interrogate many genes, proteins or binding interactions at the same time. In this case, large datasets preclude curator review of each data point, and instead receive broad overview of experimental design, controls, etc. and are presented to users clearly labeled as ‘high-throughput.’ Assays are described using the Evidence and Conclusion Ontology (ECO; evidenceontology.org; 8), which documents scientific evidence to support conclusions. Strain backgrounds denote the genetic environment in which the regulatory relationship was characterized. As with other datatypes within SGD, the 12 most commonly used strain backgrounds are recorded: CEN.PK, D273-10B, FL100, JK9-3d, RM11-1a, S288C, SEY6210, SK1, Sigma1278b, W303, X2180-1 A and Y55. Strain backgrounds not included in this list are displayed as ‘Other.’

## Regulation pages at SGD

Regulation data are displayed on locus-specific pages that list all curated regulatory relationships for a particular gene or protein. These pages are accessible via the ‘Regulation’ tab on the Locus Summary and also from the Regulation Section of the Locus Summary, where the numbers of curated regulators and targets are listed. Data are presented in tabular format on the Regulation page. Expertly written ‘regulation summaries’ have been composed for 294 genes, including all DNA-binding transcription factors in addition to other regulators. These brief, content-rich summaries include information regarding protein family classification, target genes, known regulators and biological processes affected by the regulation. Also displayed is a graph depicting the numbers of curated targets and regulators for that gene, or its gene product. On pages for DNA-binding transcription factors, which often contain characteristic domains or belong to specific protein families, information regarding protein domains and classification is included. Listed entries include the collection of domains associated with the protein based on various sources, amino acid coordinates for each domain, a domain description, a source of the classification and corresponding accession ID, and the number of *S. cerevisiae* genes that share the specified domain. Binding sites motifs as predicted by YeTFaSCo (http://yetfasco.ccbr.utoronto.ca; 9) are also presented as sequence logos for DNA-binding transcription factors. These logos graphically represent the sequence conservation of each nucleotide in the binding motif. Only high-confidence sequence motifs are included, and each logo links out to its record in the YeTFaSCo database.

For proteins that function as regulators, Regulation pages contain a ‘Targets’ Section, which includes separate tables for manually curated and high-throughput annotations. The ‘Manually curated’ table lists curated regulatory targets, while the ‘High-throughput’ table lists putative regulatory targets. The curated targets are derived from small-scale experiments that focus on one or a few genes or proteins, while the high-throughput targets are identified chiefly based on experimentally-determined presence of regulator binding in the target promoter, and/or changes in the target gene’s transcript levels in regulator mutant strains. High-throughput data are typically derived from techniques such as microarray, RNA-seq, ChIP-chip, ChIP-seq and mass spectrometry assays. Information about the assay type, cellular conditions and strain background, as well as a reference, is provided for each annotation. A GO enrichment (10) is also provided that lists the GO biological processes shared among targets listed on the page. Shared GO terms, the number of target genes that share them, and the *P*-value are all indicated in the table. A separate ‘Regulators’ Section is also included that lists regulators of the specified gene or protein; this section is organized in the same manner as the ‘Targets’ Section. The ‘Manually curated’ table lists curated regulators, while the ‘High-throughput’ table lists putative regulators based on genomic or proteomic techniques. Target and regulator data for a particular gene or protein may be downloaded as a tab-delimited text file through the ‘Download (.txt)’ buttons beneath each data table. The gene lists in each table can also be easily ported to other analysis tools within SGD via the ‘Analyze’ button. These tools include GO Term Finder (11), SPELL (12) and YeastMine (13).

**Figure 1.**
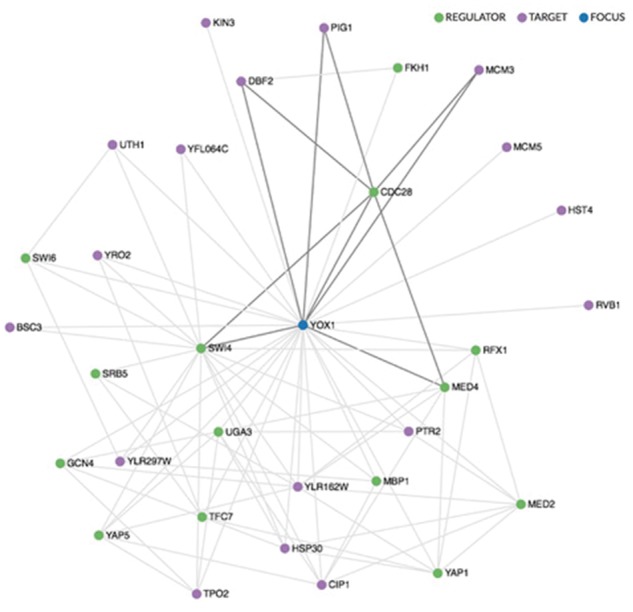
Regulatory network visualization for YOX1, shown in blue. The network is drawn using the sigma.js visualization library (sigmajs.org). Regulators are indicated in green, targets in purple. Mousing over any gene in the network highlights its local sub-network, as indicated here for CDC28, a regulator of YOX1.

Regulation pages also include a visualization of regulatory relationships in order to reflect the complexity of local regulatory networks. These displays distinguish between regulators and targets, and include a mouse-over capability that highlights internal sub-networks (Figure 1). The networks are drawn using the sigma.js visualization library (sigmajs.org).

## Future directions

Future directions for regulation curation at SGD include expansion of the revised data model to incorporate regulatory complexes in addition to single protein regulators. Future curation will also cover other types of regulators, such as RNA-binding proteins, RNA modifiers and other protein-binding entities, as well as regulation of both RNA and protein stability. In the near term, SGD will leverage existing GO annotations to various transcriptional regulation and protein modification terms, this will also include target or substrate details, resulting in additional regulatory relationships being provided on the regulation pages.

## Summary

Researchers now-a-days are facing a deluge of complex biological information. Our goal in addressing regulation in SGD reflects our central mission to aid them in sifting through these large amounts of data, comparing and contrasting relevant pieces of information that they might otherwise have missed. By combining our expertly curated regulatory relationships and written summaries with domain and binding site motifs from other resources, as described above, SGD acts as the central hub for information regarding the yeast regulatory network. The published yeast literature is curated to provide representative regulatory relationships, and controlled vocabularies are used to enhance the descriptive power of each annotation. The information will soon be distributed in defined standard formats for use in computational analysis. SGD is dedicated to maintaining the highest quality information through the application of standard representations and curation by professional Biocuration Scientists. We encourage your questions and comments. Please contact us at sgd-helpdesk@lists.stanford.edu.
